# What is the meaning of this lowercase "p"?

**DOI:** 10.1590/2176-9451.19.1.008-009.ebo

**Published:** 2014

**Authors:** José R. P. Lauris

**Affiliations:** * Associate professor, Department of Pediatric Dentistry, School of Dentistry - University of São Paulo/Bauru.

Most health professionals are resistant to statistics. There is nothing inconsistent about it: if they liked Mathematics as a profession, they would have chosen a career in the exact sciences.

Clinical dentists are not different: most of them prefer to avoid greater proximity to, and as a consequence, understanding of statistics. Undoubtedly, many statistical procedures involve complex and difficult-to-understand calculation for those who do not have expertise in this area.

It turns out that, similarly to the fact that using a car does not require knowledge of the car's intricate mechanism, electrical and electronic components, using statistics to interpret the results of a research does not require knowledge about details of the formulas that have led to these results.

An important concept in the interpretation of an article is to distinguish "descriptive statistics" and "inferential statistics."

Descriptive statistics are all procedures that aim to describe the data collected in the sample. The main statistical resources in this area are: tables, graphs, parameters such as the mean, standard deviation, correlation coefficient, absolute frequency (n), and relative frequency (%). Inferential statistics appear in the article as the p-value (probability), helping us draw conclusions from survey results obtained from the population represented by the sample.

As an example, suppose the researcher wants to investigate the effect of certain orthodontic treatment on the value of patients' SNA angle. When an article says that the pre-treatment mean SNA of a sample of 30 subjects is equal to 81.5°, this is the average value found by the researcher in the study sample. Assuming that the post-treatment mean is 80.5°, the researcher can conclude that, among the patients treated during the study, there was a mean reduction of 1° in the SNA. These results are descriptive statistical procedures. Descriptive statistics describe the data collected and, therefore, refer only to subjects in the research. This description does not involve any calculation of probability, since probabilistic error is not involved at all. Thus, it can be concluded that the treated patients had a mean reduction of 1°, because the researcher has observed this.

In a survey, however, we want to reach a conclusion not only for those who participated in the survey (sample), but also for all other subjects who did not participate in the study (population) but may need the kind of treatment studied. To make a statistical inference is to draw a conclusion for the population based on the results obtained from the sample. We intuitively make inferences in our day-to-day lives. For example, when a person has been dating for two years and decides to get married, that person is making an inference and acting upon that inference. That is because the person has evaluated those two years (sample), considered as good, and concluded that living for life together (population) will be good too. The problem is that, by making an inference (extrapolating the results from a sample to a population), we run the risk of making mistakes because we are trying to form conclusions about a larger group of elements than the one we have studied in the research. In the case of dating/marriage, we have made an inference and may be wrong: we may conclude that marriage will be good, and it may turn out not to be. In our daily lives, when we make an inference, we do not know our chances (probability) of being wrong.

What inferential statistics procedures do is simply to calculate the probability of being wrong by making an inference for the population. Therefore, in the articles, "p" simply stands for the probability of being wrong by making a certain inference about the population.

In the case of SNA, we can say that, in the treated sample, there was a reduction in SNA. The question is whether we can conclude that the treatment will alter the mean SNA of the population that can be treated by that treatment, not only the population included in the sample. If we conclude that treatment alters the population's SNA, we may be wrong: it may be that this has happened in the sample, but will not be repeated in other patients. What inferential statistics do is to calculate the probability of being wrong (p) by saying, based on the survey sample, that the treatment alters the SNA angle of the potentially treated population.

To calculate probability, statisticians use data obtained in the sample, such as mean, standard deviation, and sample size. In our example, the calculation would give as a result close to p = 0.237 (23.7%). This means that, if we conclude that the treatment changes the population's SNA, we have a 23.7% chance of being wrong. What the researcher has to do is decide, based on the statistical calculation, whether he will say that treatment alters the SNA. One researcher may find that 23.7% is a small chance of error and decide to conclude that the treatment alters the SNA; another researcher may find that the risk of error is too big and not conclude that the treatment alters the SNA. In order to have a standard for decision making, in biology in general, the adopted threshold of p value is 5% (0.05).

We name this threshold "significance level." Thus, if the p-value obtained is less than 0.05 (probability error for concluding a result for the population which is less than 5%), we conclude that what happened in the sample (SNA was changed by the treatment) is what should happen in the population. But if the p-value is greater than 0.05, we do not conclude that what happened in the sample will necessarily occur in the population. In our fictional example, as the p obtained (23.7%) is higher than 5%, we would consider it impossible to conclude that the treatment would alter the SNA. Another way of expressing the conclusion is that, if "p" is lower than the significance level (5%), we say that the difference found is statistically significant - that is, statistically speaking, there is a difference between pre-treatment and post-treatment.

As a rule, the lower the p-value is, the less the chance of error when we conclude that what has happened in the sample should happen in the population.

"Statistical tests" are the procedures for calculating the p-value. There are lots of statistical tests (e.g. t-test, analysis of variance, chi-square test, correlation test) because the best way to calculate probability varies according to several factors such as variable type, number of study groups, and whether measurements are made on the same subject or on different subjects.

No matter which test is used to calculate "p", the interpretation of "p" is the same for all types of research and every statistical test used.

Thus, every time you, the clinician, find a p-value in an article, do not be intimidated. You can interpret it without knowing what calculation was made that resulted in that value. If p-value is less than the significance level (usually 0.05), one c an extrapolate to the population the results that were found in the sample. And what was found in the sample? Simply read the results of the descriptive statistics presented.

